# The influence of the experience of trauma in childhood and the later development of psychosis. A case report

**DOI:** 10.1192/j.eurpsy.2024.1569

**Published:** 2024-08-27

**Authors:** C. Díaz Mayoral, P. Setién Preciados, E. Arroyo Sánchez

**Affiliations:** Psiquiatría, Hospital Universitario Príncipe de Asturias, Madrid, Spain

## Abstract

**Introduction:**

The increasingly well-established links between psychosis and distant traumas (often established in childhood) oppose purely neurobiological explanations. The influence of psychosocial factors on the development of a later disorder has been studied. In studies, a strong association has been found between psychosis and childhood sexual abuse, especially when sexual intercourse was involved.

**Objectives:**

A case of a patient with psychotic symptoms is presented followed by a theoretical review on the topic.

**Methods:**

A case is presented with a bibliographic review.

**Results:**

A 37-year-old woman was admitted to the Acute Hospitalisation Unit for behavioural alterations in the form of heteroaggressiveness towards family members in the context of psychopathological decompensation.

On arrival at the unit, she presented psychomotor restlessness, ideas of harm in relation to her neighbours and an attitude of referentiality, especially towards her father.

At the pharmacological level, Quetiapine 100 mg was replaced by Aripiprazole 10 mg and sleep was occasionally supported with Lormetazepam 1 mg.

Progressively her rest is normalising, she remains calm, behaviourally adequate, approachable and cooperative. She does not spontaneously allude to delusional ideation and no hallucinatory attitude is observed.

Daily individual psychotherapeutic interviews and family meetings are held with her parents, in which they refer to experiences of abandonment by her parents during her upbringing, persistent irritability and ideation of harm towards the family, which seems to be of long standing. They also report that prior to the first psychiatric admission, the patient reported being sexually abused at the age of 6 and suffered repeated physical aggression by a teacher at the age of 9. Both the patient and her parents relate the origin of the current malaise to all these events.

Upon discharge from the unit, throughout the follow-up carried out in the resource specialised in first psychotic episodes, during psychotherapeutic interviews, the feelings and emotions related to the traumatic experiences mentioned above are worked on. This therapy, associated with the pharmacological regimen previously indicated, has promoted a notable psychopathological improvement.

**Conclusions:**

A review of 46 studies in women, both inpatients and outpatients, many of whom had a diagnosis of psychosis, revealed that 48% reported having suffered sexual abuse, 48% physical abuse in childhood and 69% of them both. Among men, the figures were 28%, 50% and 59%, respectively. Childhood abuse has been shown to play a causal role in many mental health problems.

There is clear evidence that physical and sexual abuse during childhood is related to symptoms of psychosis and schizophrenia, particularly hallucinations and paranoid delusions. Also, studying possible variables, a greater severity has been observed the more intense the abuse has been.

**Disclosure of Interest:**

None Declared

